# Epitope analysis following active immunization with tau proteins reveals immunogens implicated in tau pathogenesis

**DOI:** 10.1186/s12974-014-0152-0

**Published:** 2014-09-03

**Authors:** Maj-Linda B Selenica, Hayk Davtyan, Steven B Housley, Laura J Blair, Anne Gillies, Bryce A Nordhues, Bo Zhang, Joseph Liu, Jason E Gestwicki, Daniel C Lee, Marcia N Gordon, Dave Morgan, Chad A Dickey

**Affiliations:** Department of Pharmaceutical Sciences, College of Pharmacy, University of South Florida, 12901 Bruce B Downs Blvd, Tampa, FL 33612 USA; USF Health, Byrd Alzheimer Institute, 4001 E. Fowler Avenue, MDC 36, Tampa, FL 33613 USA; Department of Molecular Immunology, Institute for Molecular Medicine, 16371 Gothard Street, H, Huntington Beach, CA 92647 USA; Institute for Memory Impairments and Neurological Disorders, University of California, 2642 Biological Sciences III, Irvine, CA 92697 USA; Department of Molecular Pharmacology and Physiology, Morsani College of Medicine, University of South Florida, 12901 Bruce B Downs Blvd, Tampa, FL 33612 USA; Department of Molecular Medicine, Morsani College of Medicine, University of South Florida, 4001 E. Fowler Avenue, MDC 36, Tampa, FL 33613 USA; Life Sciences Institute, University of Michigan, Ann Arbor, MI USA

**Keywords:** Tau, Immunogenicity, Active immunization, Neuroinflammation, Peripheral response

## Abstract

**Background:**

Abnormal tau hyperphosphorylation and its accumulation into intra-neuronal neurofibrillary tangles are linked to neurodegeneration in Alzheimer’s disease and similar tauopathies. One strategy to reduce accumulation is through immunization, but the most immunogenic tau epitopes have so far remained unknown. To fill this gap, we immunized mice with recombinant tau to build a map of the most immunogenic tau epitopes.

**Methods:**

Non-transgenic and rTg4510 tau transgenic mice aged 5 months were immunized with either human wild-type tau (Wt, 4R0N) or P301L tau (4R0N). Each protein was formulated in Quil A adjuvant. Sera and splenocytes of vaccinated mice were collected to assess the humoral and cellular immune responses to tau. We employed a peptide array assay to identify the most effective epitopes. Brain histology was utilized to measure the effects of vaccination on tau pathology and inflammation.

**Results:**

Humoral immune responses following immunization demonstrated robust antibody titers (up to 1:80,000 endpoint titers) to each tau species in both mice models. The number of IFN-γ producing T cells and their proliferation were also increased in splenocytes from immunized mice, indicating an increased cellular immune response, and tau levels and neuroinflammation were both reduced. We identified five immunogenic motifs within either the N-terminal (9-15 and 21-27 amino acids), proline rich (168-174 and 220-228 amino acids), or the C-terminal regions (427-438 amino acids) of the wild-type and P301L tau protein sequence.

**Conclusions:**

Our study identifies five previously unknown immunogenic motifs of wild-type and mutated (P301L) tau protein. Immunization with both proteins resulted in reduced tau pathology and neuroinflammation in a tau transgenic model, supporting the efficacy of tau immunotherapy in tauopathy.

**Electronic supplementary material:**

The online version of this article (doi:10.1186/s12974-014-0152-0) contains supplementary material, which is available to authorized users.

## Introduction

Accumulation of the microtubule-associated protein tau in the brain is linked to a number of neurodegenerative diseases termed tauopathies. The most common of these is Alzheimer’s disease (AD) [1]. Mutations in the MAPT gene that encodes the tau protein are known to cause some of these tauopathies, including variants of frontotemporal dementia and progressive supranuclear palsy [2,3]. Humanizing mice with transgenic insertion of these tau mutations (i.e. P301L) has provided invaluable pre-clinical tools to study tau pathogenesis. Studies using these mouse models as well as tau knockout mice have shown that removing tau could be beneficial for disease symptoms [4-7]. As a result, a number of different strategies aimed at depleting tau are being developed.

In recent years, the vaccine-based approach has shown particular promise as a method of tau reduction. Developments in tau-targeted immunotherapeutic strategies have suggested that both active and passive immunization against tau can be beneficial [8-11]. The likely reason for the efficacy of these approaches is that, reminiscent of prion propagation, tau can exit neurons, propagate to neighboring neurons, and corrupt their normal tau [12-14]. Therefore, it has been speculated that this extracellular tau is the primary target of immunological anti-tau approaches. Passive immunization in particular has shown impressive effects in pre-clinical models, and antibodies designed to target distinct abnormal tau species, such as phospho-tau, oligomeric tau, and even misfolded tau, have all proven effective in mice [4,10,11,15-19].

While active immunization paradigms against self-proteins will likely not be therapeutically relevant, they have served as proof-of-principle for the vaccination approach in general. Indeed, active immunization of mice with tau peptides produced tau antibodies that could cross the blood-brain barrier, (BBB) reduce pathological tau [1,20], and rescue functional impairments in tau transgenic mice [9,10,21]. So far, however, these peptides have all been generated based on pathological relevance or predicted immunogenicity using algorithms; there has not been an epitope map produced to identify the strongest immunogens in the tau sequence. To fill this void, we vaccinated both tau transgenic and non-transgenic mice with human wild-type tau (Wt, 4R0N) protein or human P301L tau (4R0N). Not only was tau pathology found to be reduced after vaccination, but using the anti-sera we created an epitope map via an innovative peptide array approach that revealed the evolutionary emergence of a novel tau immunogen found only in primates. In addition, we have determined that transgenic and wild-type mice produce anti-sera with distinct epitope profiles, depending on whether or not they were vaccinated with wild-type tau or mutant P301L tau. These findings suggest that specific tau regions are highly immunogenic and may aid in the development of improved tools to study the effects of vaccination strategies against tauopathies.

## Materials and methods

### Animals

The rTg4510 mouse breeding was performed by crossing the parental human P301L tau mutation and tetracycline-controlled transactivator (tTa) phenotypes as originally described in [5]. Age-matched, non-transgenic littermates were included in the study (in-house breeding). Animal procedures were consistent with the recommendations of the National Research Council’s “*Guide for the Care and Use of Laboratory Animals*”, and were approved by the University of South Florida animal care and use committee (IACUC).

### Protein expression and purification

The human wild-type 4R0N Tau or human P301L tau (4R0N) were expressed with N-terminal 6xHis-tags using the pET28 vector system (EMD Millipore, Darmstadt, Germany). The plasmids were transformed into One Shot® BL21 Star™ (DE3) *E. coli* cells from Invitrogen (Invitrogen, Grand Island, NY, USA) and then grown in 1.0 L LB media under kanamycin selection (50 μg/mL). Expression was induced at optical density 0.7 with 1.0 mM isopropyl-β-D-thiogalactopyranoside and growth continued for 3 hours at 37°C with 250 rpm shaking for aeration. Cells were pelleted by centrifugation at 4,000 *g* for 15 minutes at 4°C and resuspended in 35 mL lysis buffer (500 mM NaCl, 10 mM Immidazole, 1.0 mM phenylmethylsulfonyl fluoride, 10 mM Tris/HCl, pH 8.0). Resuspended pellets were stored at −80°C.

Cells were thawed on ice and lysed by sonication. Lysate was clarified by centrifugation at 50,000 *g* for 30 minutes at 4°C. The supernatant was then loaded on a pre-charged Ni-NTA column (Qiagen, Valencia, CA, USA) and washed with 50 mL wash buffer (500 mM NaCl, 10 mM Immidazole, 10 mM Tris/HCl, pH 8.0). The 6xHis-tagged proteins were eluted in 20 mL elution buffer (500 mM NaCl, 250 mM Immidazole, 10 mM Tris/HCl, pH 8.0) and then concentrated to 2 mL using Millipore Amicon Ultracel-10 centrifuge tubes (EMD Millipore). The concentrated samples were then loaded on a HiLoad 16/600 Superdex 200 pg column (GE Healthcare, Pittsburgh, PA, USA) that was pre-equilibrated in size exclusion buffer (500 mM NaCl, 0.5 mM EDTA, 0.1 mM DTT, 10 mM Tris/HCl, pH 8.0). Eluted fractions were pooled, concentrated, and dialyzed into PBS (pH 7.4) overnight. Samples were stored at −80°C.

### Vaccination procedure and tissue collection

**Figure 1:**
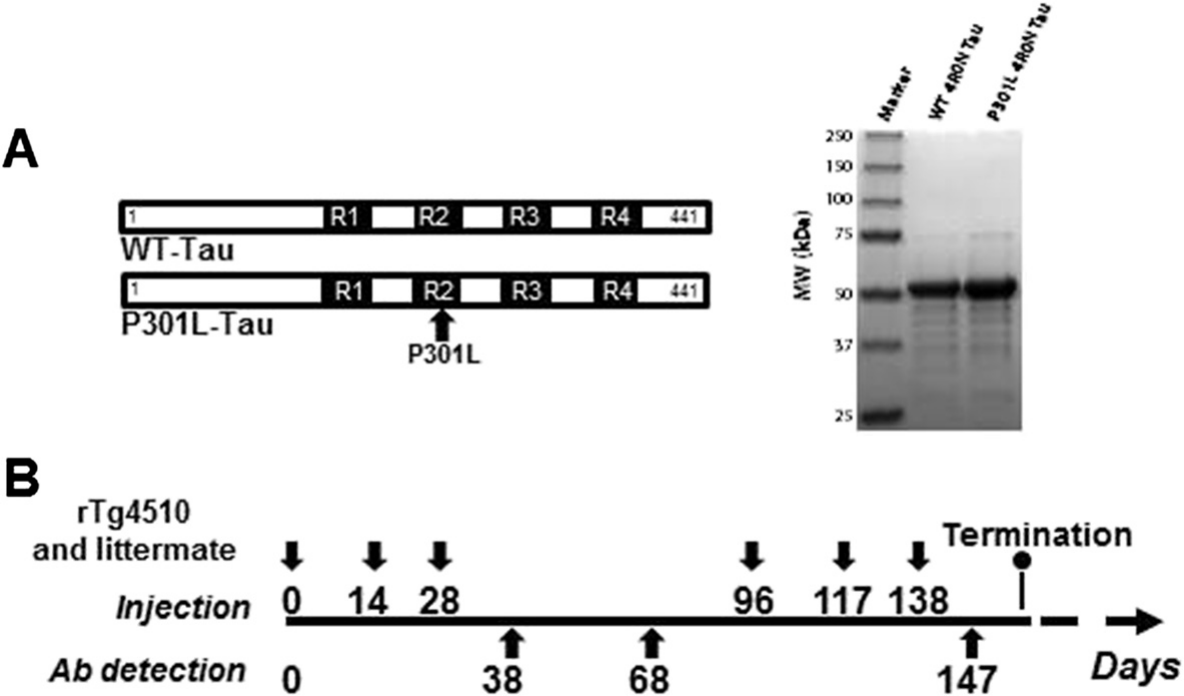
**Structure and purification of recombinant tau proteins followed by the immunization paradigm.** **(A)** Schematic presentation of the sequence and commassie stain of recombinant full length tau protein (wild-type (Wt)-tau, 4R0N) or tau mutated at P301L (P301L-tau, 4R0N). **(B)** Non-transgenic and rTg4510 mice were immunized three times in alternating weeks, followed by a 10-week resting period, then boosted three times every 3 weeks. After the vaccination period, tissue and blood specimens were analyzed for cellular immune responses and neuropathological changes. Ab, antibody; MW, molecular weight.

Male and female transgenic rTg4510 mice (5 months old; n = 12) and their non-transgenic littermates (n = 12), subdivided into six groups (n = 4 per group), were immunized with either protein or PBS (control group, Table 1). Mice were injected subcutaneously with a 100 μg tau antigen formulated with Quil-A adjuvant (20 μg per mouse). All groups received three injections in alternating weeks and were boosted an additional three times (3 weeks apart) after a 10-week resting period with appropriate antigen (Figure 1). Sera were collected at days 38, 68, and 147, and were used to measure anti-tau antibody responses. Mice were sacrificed with somnasol (0.078 mg/ml pentobarbital, 0.01 mg/ml phenytoin sodium) at day 9 after the last immunization. Spleens were removed and placed in 5 mL RPMI1640 (Invitrogen). Blood was drawn intracardially and stored at 25°C for 1 hour, placed at 4°C overnight, and then centrifuged at 4,000 rpm for 10 minutes. Serum was collected and centrifuged again at 7,000 rpm for 10 minutes. Brains were collected following transcardial perfusion with 0.9% normal saline solution. Fixed mouse brains were cryoprotected in successive 24-hour incubations of 10%, 20%, and 30% sucrose solutions and then sectioned as described previously [22].

**Table 1:** **Immunization paradigm and group assignment**

**Group**	**Mice**	**Immunogen**	**Number of mice**	**Route**	**Adjuvant**
1	Littermate	Wt-Tau	4	Subcutaneous	Quil A
2	rTg4510	Wt-Tau	4	Subcutaneous	Quil A
3	Littermate	P310L-Tau	4	Subcutaneous	Quil A
4	rTg4510	P310L-Tau	4	Subcutaneous	Quil A
5	Littermate	PBS	4	Subcutaneous	Quil A
6	rTg4510	PBS	4	Subcutaneous	Quil A

rTg4510 mice (n = 12) and non-transgenic littermates (n = 12) were separated into 6 groups (n = 4 per group). Each mouse was subcutaneously injected with a mix solution of 1.1 mg/ml of each protein formulated in 2.0 mg/ml Quil A adjuvant. Each groups respective immunogen, immunization route, and adjuvant used are indicated. PBS, phosphate-buffered saline; Wt, wild-type.

### Tau peptide microarray

The tau peptide microarray was designed using 15-mer peptides with four amino acid overhangs, spanning the full sequence of PNS-tau (P10636-9) for a total of 187 tau peptides. Empty spots were used as negative controls. The arrays were printed on single microscope slides in triplicate (Jenrin Peptide Technologies, Berlin, Germany). Binding was tested per manufacturer’s protocol using plasma, diluted 1:100 in binding buffer (PBS/0.1% Tween/2% bovine serum albumin) from animals treated with either Wt-tau or P301L tau protein. Binding was detected using a 1:500 titer of HiLyte Fluor 555-labeled goat anti-mouse IgG antibody (Anaspec, Fremont, CA, USA) in binding buffer. Arrays were scanned at a fluorescence emission of 532 nm using a GenePix 4100A Microarray Scanner (Molecular Devices, Sunnyvale, CA, USA) and raw intensities adjusted by background subtraction (morphological closed followed by opening) using the scanner software (GenePix Pro 7, Molecular Devices, Sunnyvale, CA, USA). Binding was defined as peptides with fluorescence intensity greater than 3 standard errors above the mean of the slide intensities. In order to identify false positives, a microarray was incubated with the antibody alone. No signal was observed, so all peptides were included in the analysis.

### Detection of anti-tau antibody responses

Mice were bled from the submandibular region at days 0 (pre-bleed), 38, 68, and 147 of the immunization schedule. The titers of anti-tau antibodies were determined by ELISA as described previously [23], with minor modifications. Briefly, 96-well plates (Immunol 2HB; Fisher Scientific, Pittsburgh, PA, USA) were coated with 100 μL of 10 μg/ml Wt-tau and P301L-tau (pH 9.7, overnight at 40°C). Wells were washed and blocked with 3% non-fat milk in 1xTris-Tween buffered saline overnight, and then 100 μL of pooled sera from immunized mice were added to the wells at different dilutions. After incubation and washing, HRP-conjugated goat anti-mouse IgG (1:2,500, Jackson ImmunoResearch Laboratories, West Grove, PA, USA) was used as a secondary antibody. Plates were incubated and washed, and the reaction was developed by adding 3,3′,5,5′ tetramethylbenzidine (Pierce, Rockford, IL, USA) substrate solution and stopped with 2 M H_2_SO_4_. The optical density was read at 450 nm (Biotek, Synergy HT, Winooski, VT, USA). Endpoint titers of antibodies were calculated as the reciprocal of the highest sera dilution that gave a reading twice above the cutoff. The cutoff was determined as the titer of pre-immune sera at the same dilution. The above procedure was repeated a total of three times, with inter-assay variations of only 6 to 10%. Average data of three ELISAs are presented. For determination of endpoint titers, sera were serially diluted up to 1:102,000 from an initial dilution of 1:3,000. HRP-conjugated anti-IgG1, IgG2a^b^, IgG2b and IgM specific antibodies (1:2000; Bethyl Laboratories, Inc., Montgomery, TX, USA) were used to characterize the isotype profiles of anti-tau antibodies in individual serum (day 38) at dilution 1:1,000 (plates were coated with Wt-tau protein). The optical density at 450 nm values for pre-bleed (day 0) samples were subtracted from the day 38 samples.

### Detection of cellular immune responses

T-cell proliferation analysis was performed in splenocyte cultures from individual animals using [^3^H]-thymidine incorporation assays, as previously described in [23]. Splenocytes were re-stimulated *in vitro* with Wt-tau, P301L-tau or irrelevant proteins. Cells were first incubated for 72 hours, then 1 μCi of [^3^H]-thymidine (Amersham Biosciences, Piscataway, NJ, USA) was added to each well for 16 to 18 hours. Cells were harvested using the Tomtec Mach III harvester (TOMTEC LifeSciences, Hamden, CT, USA), and [^3^H] thymidine uptake (cpm) was counted on a Microbeta 1450 Trilux scintillation counter (Wallac, Pelkin-Elmer, Waltham MA, USA). The stimulation index was calculated as previously described in [23]. The same splenocytes were also used to assess T-cell activation through IFNγ ELISPOT assays (BD Pharmingen, San Jose, CA, USA), as previously described [24]. Splenocytes from individual mice were re-stimulated with Wt-tau, P301L-tau, or irrelevant proteins. Spots were counted using a CTL-Immunospot S5 Macro Analyzer (Cellular Technology Ltd., Shaker Heights, OH, USA). The difference in the number of spot forming colonies per 10^6^ splenocytes that were re-stimulated with vaccine-related proteins and those found in 10^6^ splenocytes re-stimulated with irrelevant proteins was calculated. All proteins were used at 10 μg/ml.

### Immunohistochemical analysis

Immunohistochemistry was performed on free-floating sections as recently described in [25]. A series of six sections per animal were incubated with each primary antibody overnight at room temperature. The following primary antibodies were used for immunohistochemistry: anti-total tau (H150, Santa Cruz Biotechnologies, Dallas, TX, USA); anti-phospho-tau at Serine 396 (pS396), anti-phospho-tau at 199/202 (pS199/202, Anaspec, Fremont, CA, USA); anti-phospho-tau at Serine 202 and Threonine 205 (biotinylated AT8, ThermoScientific, Waltham, MA, USA); anti-Paired Helical Filament-1 tau (PHF1/pS396-404, kind gift from Dr Peter Davies, Albert Einstein College of Medicine, Yeshiva University); anti-Cluster of Differentiation 45 (CD45, ThermoScientific); anti-Cluster of Differentiation 11b (CD11b, AbD Serotec, Raleigh, NC, USA); anti-glial fibrillary acidic protein (GFAP; DAKO, Carpinteria, CA, USA); anti-major histocompatibility complex II (BD Biosciences, San Jose, CA, USA). HRP- and biotin-conjugated secondary antibodies were obtained from VectorLabs (Burlingame, CA, USA), while conjugated AlexaFluor594/488 secondary antibodies were purchased from Invitrogen. Color development was performed using 0.05% 3,3′-diaminobenzidine (Sigma, St Louis, MO, USA) enhanced with 0.5% nickelous ammonium sulfate (J.T. Baker Chemical Company, Phillipsburg, NJ, USA). Gallyas histology was performed as described in [26] using sections that were pre-mounted on slides and then air dried for a minimum of 24 hours.

Stained sections were imaged using a Zeiss Mirax150 digital scanning microscope (Carl Zeiss MicroImaging, GmbH Clincial, 07740 Jena, Germany). For quantification, regions of anterior cortex, hippocampus and the entorhinal cortex were analyzed by using hue, saturation and intensity for each image field. Thresholds for object segmentation were established with images of high and low levels of staining to identify positive staining over all intensity levels within the study. These limits were held constant for the analysis of every section in each study according to Gordon and colleagues [27]*.*

### Statistical analysis

Statistical analyses were performed using Student’s *t*-test or one-way analysis of variance followed by Fischer’s LSD *post hoc* means comparison test using Stat View software version 5.0 (SAS Institute Inc., Cary NC, US). Graphs were generated using GraphPad Prism 5.0 (La Jolla, CA, USA). The average% positive area value measured by immunohistochemistry was normalized to the PBS controls.

## Results

### Active immunization induces robust anti-tau antibody response

**Figure 2:**
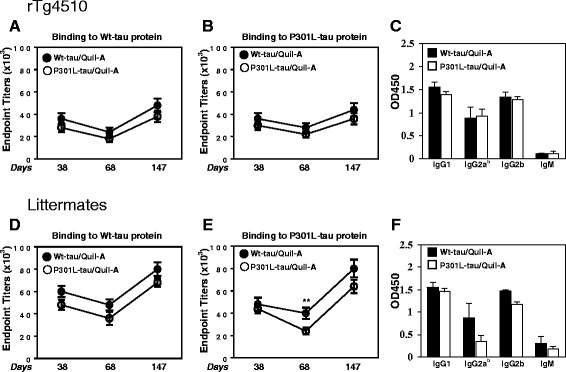
**Immunizations with Wt-tau and P301L-tau proteins formulated in Quil-A induced robust titers of anti-tau antibody in rTg4510 and littermate mice.** Mean endpoint antibody titers specific for wild-type (Wt)-tau **(A,D)** and P301L-tau **(B,E)** were evaluated in pooled sera of mice (n = 4 per group) at days 38, 68, and 147. Endpoint titers of antibodies represents the highest sera dilution (1:108,000 from and initial dilution of 1:3,000) with two-fold signal above the cutoff. The cutoff was determined as the titer of pre-immune sera at the same dilution. ELISAs were repeated three times and average ± SD of three ELISAs is presented (student’s *t*-test was performed, **P < 0.01). **(C,F)** Individual sera of immunized rTg4510 mice and littermates at day 38 (1:1,000) were analyzed and IgG anti-tau antibody of IgG1, IgG2a^b^, and IgG2b isotypes were detected. Error bars indicate the average ± SD (n = 4).

The beneficial effects of tau immunotherapy have been previously demonstrated by several research laboratories [8,10,11,19,21,28]. To date, there have been no investigations of immunogenicity of various forms of tau protein or subsequent humoral and cellular immune responses following vaccination. Therefore, 5-month-old transgenic rTg4510 mice with existing pathology (n = 12) and non-transgenic littermates (n = 12) were immunized as shown in Figure 1 and Table 1. The rTg4510 mouse model was chosen because it is currently the state-of-the-art mouse model for tauopathy. It is one of the only models to produce the pathological conformations necessary to induce Gallyas silver positive staining, which is a common benchmark for identifying tangle pathology in humans. In addition, these mice develop pathology in the forebrain, which begets neuronal loss and glial activation [5,26,29]. Pooled sera from mice bled at days 38, 68, and 147 were analyzed via ELISA. On day 38, both transgenic and non-transgenic mice showed robust antibody titers specific to each tau form (Wt or P301L). These antibodies remained present during the rest period (day 68), suggesting a persistent antigen-specific immune response. The antibody titers increased following booster injections on day 147. During the experimental course, no significant difference in titer quantities were measured between Wt-tau or P301L-tau in vaccinated rTg4510 (Figure 2A,B) and littermate mice (Figure 2D,E). After immunizations of mice with both proteins, levels of anti-tau titers are higher in littermates compared to P301L mice, which could be related with the mechanism of self-antigen tolerance. The B cell receptor can recognize native foreign and self-molecules equally well; however, CD4^+^ T helper cell receptors recognize mostly foreign, but not self-peptides presented by self-major histocompatibility complex class II molecules. Therefore, it is not surprising that the levels of anti-tau titers are low in rTg4510 mice since the tau protein is a self-antigen for these animals. The generation of a potent antibody response to tau vaccination requires the breaking of natural tolerance to self-antigens. Formulation of antigen in strong adjuvant helps to breake the tolerance although the response could be still lower compared with foreign antigen (in this case human tau in non-transgenic mice). As expected, the control PBS injection did not elicit antibody production in these mice (data not shown).

Analyzing the isotype of antibody produced, we showed that injection of either the wild-type or P301L recombinant tau induced in all immunized mice an almost equal IgG1, IgG2a^b^, and IgG2b isotype response, but a much lower IgM response (Figure 2C,D). Overall, the average abundance of the generated isoforms was independent of genotype and antigen and ranked as follows: IgG1 > IgG2b > IgG2a^b^ > IgM.

### Immunization with tau proteins induces a strong cellular immune response

**Figure 3:**
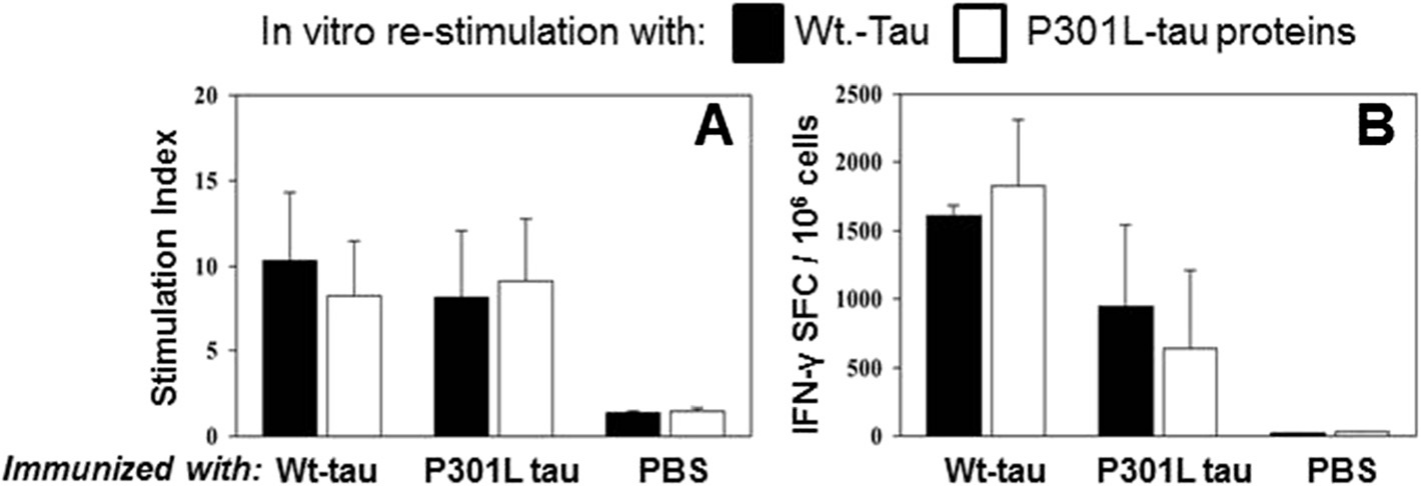
**Cellular immune responses specific to different tau proteins were detected in rTg4510 mice following immunizations with wild-type (Wt)- or P301L-tau proteins. (A)** Proliferation of T cells is detected by [3H]-thymidine incorporation assay in splenocyte cultures obtained from experimental and control animals and expressed as stimulation index. **(B)** Number of IFN-γ producing cells (spot formatting cells, SFC) is detected by ELISPOT assay in splenocyte cultures obtained from immunized rTg4510 mice. Bars represent average ± SD (n = 4).

To discern the levels of T cell proliferation following immunization we employed a [^3^H]-thymidine incorporation assay [23] which measured the amount of [^3^H]-thymidine incorporated into cells as they proliferated. Splenocytes from experimental and control groups of rTg4510 mice vaccinated with the Wt- or P301L-tau induced strong T-cell proliferation, though the control PBS did not (Figure 3A). These strong cellular immune responses in all vaccinated mice were confirmed by measuring the number of IFN-γ producing T cells (Figure 3B). PBS-injected mice exhibited only background levels of spot-formulating cells after *in vitro* re-stimulation with both proteins (Figure 3B). This data demonstrates that immunization of rTg4510 mice with each tau protein elicits a robust and specific anti-tau antibody generation, followed by a strong cellular immune response.

### Identification of tau immunogenicity

**Figure 4:**
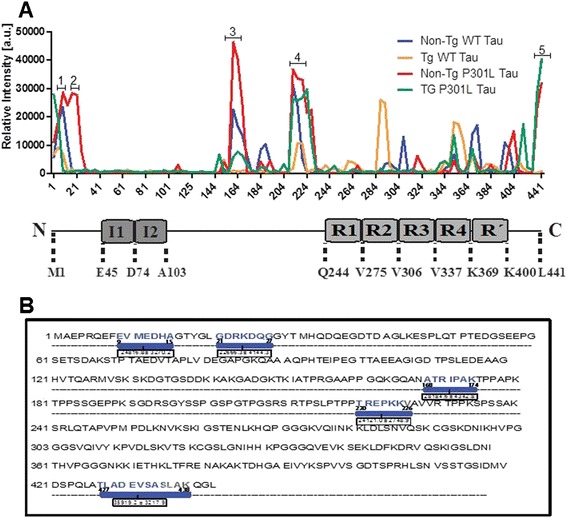
**Immunogenicity of tau protein detected by spot array. (A)** The profile of the epitope mapping experiment where the fluorescence intensities at a wavelength of 532 nm, after background subtraction, for each animal group tested in duplicate is shown. The domain organization of human tau in the central nervous system (441 residues, htau40) containing the I1-I2 inserts on the N-terminal and the R1-R4 repeats is shown. The domain boundaries are labeled by the residue numbers. Five peaks displayed the highest fluorescence intensity (peak 1-5). **(B)** Amino acid sequence of 4R2N expressed in the central nervous system. Blue colored amino acids represent the five peptide sequences with the highest immunogenicity identified in the spot array. Each highlighted peptide sequence corresponds to the peak 1-5 in (A). Average fluorescence intensity ± SEM is shown for each sequence. a.u., Area under; Tg, transgenic; WT, wild-type.

The above findings suggest that regions of tau sequence can elicit both humoral and cellular activation. As tau immunotherapies continue to be a promising approach in neurodegenerative disease, it is attractive to pursue studies where tau epitopes are targeted based on their sequence immunogenicity profile. To identify immunogenic tau peptides responsive to the vaccination paradigm, we performed tau epitope mapping by utilizing a novel peptide microarray composed of 15-mer peptides covering the longest isoform of tau expressed in the peripheral nervous system [30]. These tau peptides were assembled with four amino acid overlaps, and each presented in triplicate on glass slides. Binding of sera from animals vaccinated with Wt-tau or P301L-tau to these peptide arrays was measured using fluorescent anti-mouse IgG antibody. Negative controls consisted of fluorescent anti-mouse IgG antibody alone. As expected, the negative controls bound nonspecifically to very few peptides, which were excluded from subsequent analyses. In contrast, sera from Wt-tau and P301L-tau immunized rTg4510 and non-transgenic animals bound to a number of tau-derived peptides (Figure 4A, Additional file 1: Table S1). Analysis of the fluorescence intensity measured in this assay identified ten epitopes with high immunogenicity (Additional file 2: Table S2). Further analyses ranked the sequences with the highest florescence intensity, as outlined in Figure 4B. These motifs lay within the N-terminal region (9-15 and 21-27 amino acids), proline rich region, microtubule binding region (168-174 and 220-228 amino acids) and the C-terminal region (427-438 amino acids) (Figure 4). The 2 N-insert tau region displayed low binding activity toward any of the tau forms tested in the array (Figure 4A). The peptide microarray findings suggest that these sequences can elicit antigen-induced peripheral immune activation.

### Efficacy of anti-tau immunization in neuroinflammation

**Figure 5:**
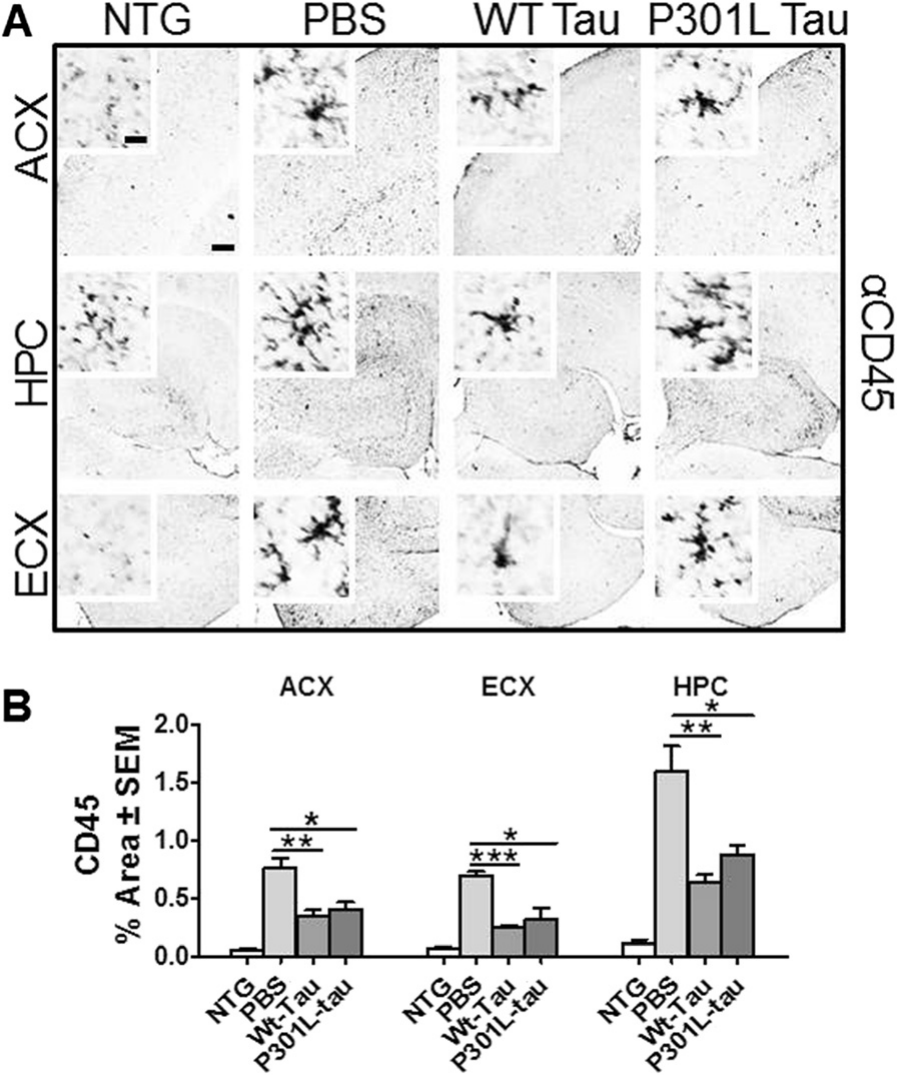
**Reduced activated microglia burden following immunization with wild-type (WT)- or P301L-tau proteins. (A)** Representative micrographs of brain regions (anterior cortex (ACX), hippocampus (HPC), and entorhinal cortex (ECX)) immunostained for CD45 microglia marker. **(B)** Percent positive area analysis of the brain regions demonstrated significant reductions in CD45 microglia burden in both Wt-tau and P301L-tau immunized rTg4510 animals. Immunized non-transgenic animals displayed low levels of activated microglia, hence pooled values were graphed. One way analysis of variance, * *P* < 0.05, ** *P* < 0.01, *** *P* < 0.001. Scale bar is 200 μm, inset 20 μm. NTG, non-transgenic; PBS, phosphate-buffered saline.

**Figure 6:**
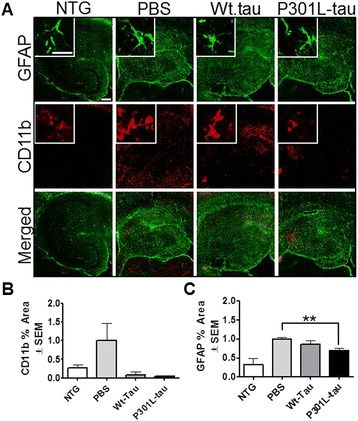
**Reduced inflammation following vaccination with wild-type (Wt)- or P301L-tau proteins. (A)** Representative micrographs of hippocampal region positive stain for glial fibrillary acidic protein (GFAP; green) and CD11b (red) in immunized rTg4510 and non-transgenic (NTG) littermates. **(B,C)**. Quantification of percent positive area of hippocampus demonstrated significant reduction in the levels of GFAP following P301L-tau but not Wt-tau immunization. No significant reduction in CD11b immunoreactivity was observed following rTg4510 mice immunization compare to phosphate-buffered saline (PBS)-injected littermates. Immunized non-transgenic animals displayed low levels of GFAP and CD11b immunoreactivity (pooled values). Values are normalized to the PBS control group and student’s *t*-test was performed, **P < 0.01. Scale bar is 200 μm for regions, 20 μm for insets.

We have previously shown that rTg4510 mice display induced levels of inflammation with age [26]. To assess the neuroinflammatory response following immunization, and to see whether a tau-induced immune response in the periphery can translate into changes in the central nervous system neuroinflammatory mileu, we investigated the state of microglial (anti-CD45, anti-CD11b) and astrocytic activation (anti-GFAP) by a immunohistochemical approach, using markers associated with microglia and astrocyte activation [31,32]. Quantification of percent positive area showed significant CD45 reduction in the anterior cortex (Wt- and P301L, *P* < 0.01 versus *P* < 0.05, respectively), hippocampus (*P* < 0.005 and *P* < 0.05, respectively), and entorhinal cortex (*P* < 0.0001 and *P* < 0.05, respectively). Non-transgenic mice displayed quiescence microglia morphology (insets in Figure 5A) and low levels of CD45 immunoreactivity (Figure 5B). Furthermore, vaccinated rTg4510 mice displayed reduced levels of CD11b-positive microglia in the brain (Figure 6A, red fluorescent), reaching levels comparable to those measured in immunized non-transgenic mice (Figure 6B). In agreement with previous data, rTg4510 mice have augmented microglia burden [26] as shown by the increased CD11b levels in PBS-injected mice. Stain for GFAP immunoreactivity (Figure 6A, green fluorescent) showed significant GFAP signal reduction in P301L-tau immunized mice (*P* < 0.0066), while only slight reductions were observed in the Wt-tau immunized mice compared to the PBS-injected animals. Non-transgenic mice showed low levels of astrocyte activation in the brain (Figure 6C). Overall, vaccination of rTg4510 mice resulted in reduced neuroinflammatory milieu with no effect present in immunized non-transgenic littermates.

### Immunization against Wt- or P301L-tau protein significantly reduces pathology in rTg4510 mice

**Figure 7:**
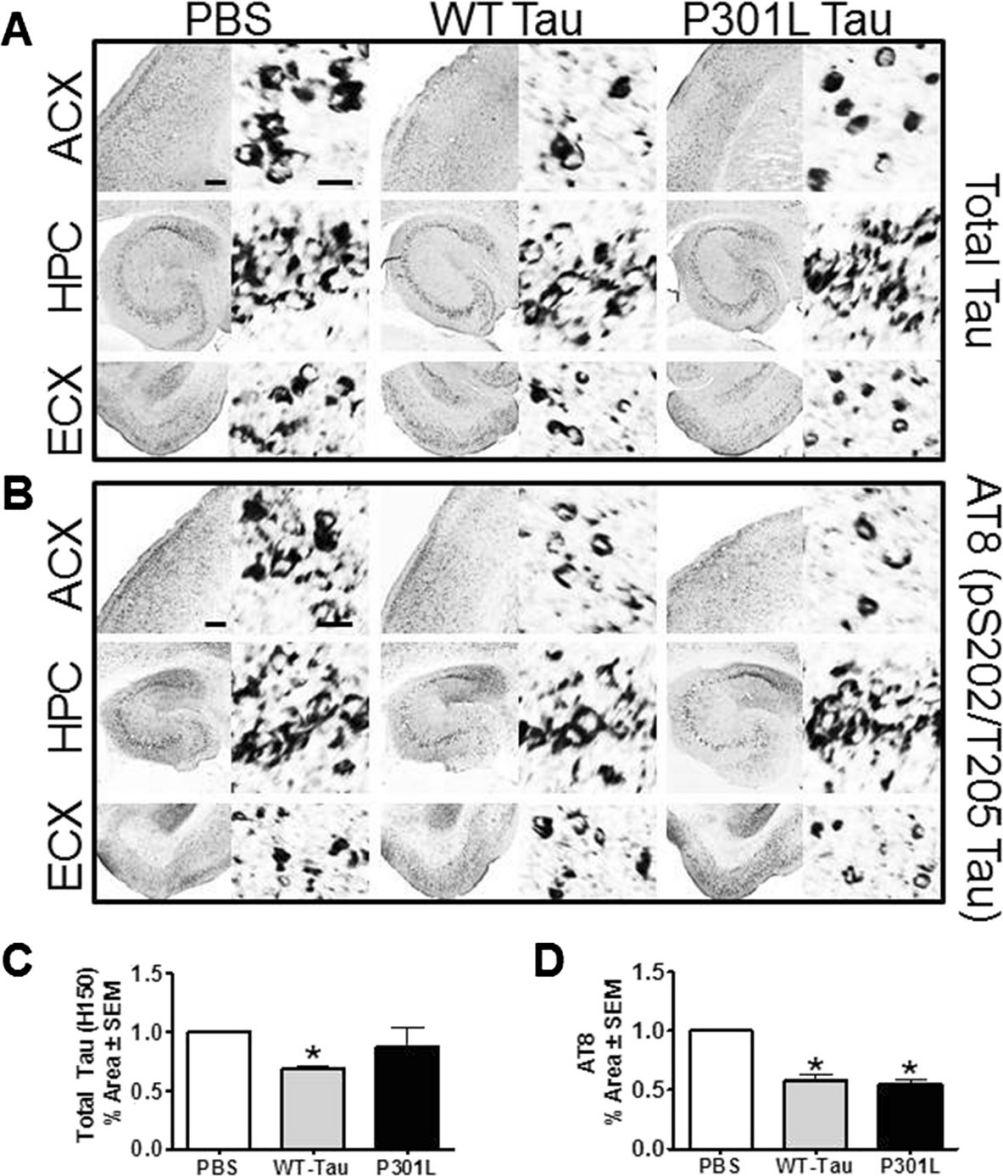
**Vaccination with wild-type (WT)- or P301L-tau proteins reduced tau pathology. (A)** Micrographs of brain regions (anterior cortex (ACX), hippocampus (HPC), and entorhinal cortex (ECX)) immunostained for total-tau (H150) and **(B)** AT8 (pS202-Thr205). **(C)** Analysis of percent positive area in each region demonstrated significant reduction of total tau in the animals immunized with Wt-tau, while **(D)** reduction in AT8 (pS202-Thr205) tau levels was observed in both groups as compared to phosphate-buffered saline (PBS)-injected animals. Values are normalized to PBS controls and student *t*-test was performed, *P < 0.05. Scale bar is 200 μm for regions, 20 μm for insets.

Finally, we investigated the impact of immunization on tau pathology in transgenic mice by performing immunohistochemistry (Figure 7 and Table 2). Staining of brain tissue showed significant reductions in total tau (H150, Figure 7C) following vaccination with Wt-tau but not P301L-tau injected rTg4510 mice (Table 2; *P* = 0.0179 and *P* = 0.6452, respectively). As indicated in Figure 7A,D, we observed significant reduction in pS202-Thr205 tau levels (AT8) in transgenic mice vaccinated with both Wt-tau and P301L-tau (Table 2; *P* = 0.0295 and *P* = 0.0377, respectively). The results from tissue analysis from several phospho-tau antibodies and aggregated tau (Gallyas stain) are summarized in Table 2. Consistent with previous studies [33], the immunohistochemical analyses suggest a beneficial effect of tau vaccination regarding reduction in cerebral tauopathy and inflammation in the rTg4510 mouse model.

**Table 2:** **Immunohistochemical changes measured in immunized mice**

	**Brain % positive area (average ± SEM)**	***P*** **-values (Student** ***t*** **-test)**
**H150 (total tau)**		
Wt-tau	0.68 ± 0.03*	0.0179*
P301L tau	0.87 ± 0.17	0.6452
**AT8 (pS202-Thr205)**		
Wt-tau	0.58 ± 0.05*	0.0295*
P301L tau	0.55 ± 0.03*	0.0377*
**pS199/202**		
Wt-tau	0.59 ± 0.04	0.0721
P301L tau	1.29 ± 0.19	0.3600
**pS396**		
Wt-tau	0.96 ± 0.06	0.8089
P301L tau	0.99 ± 0.04	0.9700
**Gallyas**		
Wt-tau	1.27 ± 0.06	0.4596
P301L tau	0.81 ± 0.06	0.5839
**CD45**		
Wt-tau	**0.4 ± 0.03**	0.0001*
P301L tau	**0.51 ± 0.03**	0.0001*
**CD11b**		
Wt-tau	0.12 ± 0.07	0.1202
P301L tau	0.04 ± 0.02	0.0550
**GFAP (HPC)**		
Wt-tau	0.85 ± 0.1	0.2977
P301L tau	**0.7 ± 0.05**	0.0066*

The ratio of percent positive area values for each immunized group to the phosphate-buffered saline (PBS)-injected mice is presented for total tau, p-tau and inflammatory markers (average ± SEM). Values in bold and asterisk indicate markers that are significantly reduced by immunization. Statistical analyses were performed by student’s *t*-test, *P < 0.05. GFAP, glial fibrillary acidic protein; HPC, hippocampus; Wt, wild-type.

## Discussion

Immunotherapeutic approaches to treat tauopathies are being intensively pursued by both academia and industry. While active immunization strategies will likely not be a viable therapeutic approach due to concerns about auto-immunity and micro-hemorrhage, it could be useful for understanding the interface between the immune response and tau pathogenesis. Using active immunization against the 4R0N wild-type or P301L tau, we determined the most immunogenic epitopes in the central nervous system tau isoform. One of these epitopes (amino acids 21-27 GDRKDQG), a sequence that introduces a caspase cleavage DXXD motif and is linked to tau pathogenesis, is exclusively found in tau from primates, suggesting that the evolutionary insertion of this fragment could be an important reason for the emergence of tau pathogenesis in humans. In addition, we show that while non-transgenic and tau transgenic mice do produce some similar epitopes to wild-type and mutant human tau, they also produce anti-sera to unique epitopes. Subcutaneous injections of Wt-tau and P301L-tau proteins in the rTg4510 mouse model and non-transgenic littermates resulted in equal and robust antibody titers and cellular immune response specific to both antigens, and active vaccination with either tau protein similarly reduced tau pathology and neuro-inflammation in tau transgenic mice, though not to the extent expected based on previous work. One possibility may be that peripheral immune cells are the major contributor to the beneficial effects of vaccination more than resident microglia, as previously described [34-36], but, perhaps by improving antigen design based on antigen profiling data, the efficacy of vaccination strategies can be advanced. Using a peptide microarray assay, we identified several tau regions with similar immunogenicity in non-transgenic and rTg4510 mice immunized with either Wt or mutant P301L tau. These common active epitopes were found throughout the protein: two were located in the N-terminal (9-15 and 21-27 amino acids; EVMEDHAG, GDRKDQ), one in the proline rich region (168-174 and 220-228 amino acids; ATRIPAK, TREPKKV) and one at the extreme C-terminal region (427-438 amino acids TLADEVSASLAK). Each of these immunogenic regions was of particular interest because of their biological significance.

The 9-15 amino acids, 21-27 amino acids, and 427-438 amino acids sequences are within regions that are cleaved by caspases [37,38]. *In vitro*, N-terminal cleavage of tau has been suggested to occur at the aspartic acid position 13 (VMED^*^-), but other potential cleavage sites are also present (for example, the DXXD motif within the 21-27 amino acids epitope [39]). Although the N-terminal truncation decreases tau polymerization *in vitro*, studies have suggested that the Alz50-Tau66 conformation, which requires the N-terminus, can stabilize existing filaments and contribute to tangle maturation in AD [37]. The *in vitro* D421 truncation of tau (DMVD*-) can nucleate tangle formation [40-42]. Moreover, tau that is released from neurons has been found to be caspase cleaved. The accumulation of tau cleaved at amino acid 421 into tangles has been demonstrated in the brains of several animal models [43,44] and in AD patients [45]. Combined with our new data, it suggests that tau outside of neurons and accumulating into tangles may lack a key immunogenic epitope that could assist in its clearance. However, when tau is phosphorylated at S422, its caspase cleavage is blocked and this phospho-tau species is also found in tangle pathology. In fact, a previous study showed that active immunization targeting pS422 in tau transgenic mice led to an immune response and reduction of tau pathology followed by significant cognitive improvement [21]. These findings suggest that multiple factors can contribute to tau pathology, and the interface between the biology and immunological response to these distinct tau species could be essential to designing improved treatments targeting tau.

The tau immunogens within the proline-rich region at 168-174 amino acids and 220-226 amino acids also are near a number of consensus sites for known tau kinases: microtubule affinity regulating kinase (MARK), mitogen activated protein kinase (MAPK), cAMP-dependent protein kinase and glycogen synthase kinase 3 (GSK3). Systemic studies suggests that phosphorylation of tau by proline-directed kinases (MAPK and GSK3) in the regions adjacent to the repeat domain (R1-R4) had a weak negative effect on tau-microtubule interactions as well as tau aggregation into paired helical filaments (PHF) [46,47]. However, immunization of K257/P301S mice with a mixture of phopho-Tau195-203, 207-220 and 224-238 peptides demonstrated a reduction in levels of phospho-tau and neurofibrillary tangle burden [28], indicating beneficial effects of targeting this region. Interestingly, none of the five most potent immunogens were located within the microtubule binding domain of tau, despite this being the region where most disease-causing mutations lie and the region that is the most active with regard to function and aggregation. However, some of the less potent epitopes did lie within the microtubule binding domain. In particular, we found that only vaccinated tau transgenic rTg4510 mice produced antisera with activity against epitopes containing consensus sites for MARK2 (amino acids 292-302 and 356-366). These sites are known to be very important for tau function and pathogenicity. When phosphorylated at these sites, tau attachment to microtubules was inhibited and had a greater propensity to aggregate [46,48]. Tau phosphorylated at these sites is also found in tau tangles [49]. It is possible that when these sites are phosphorylated, their immunogenicity is changed, contributing to tau accumulation. It also suggests that over-expression of mutant tau can expose distinct epitopes that could be exploited to treat specific tauopathy subtypes.

Immunotherapy targeting tau is under intensive investigation. Understanding the interface between tau and the immune system could prove invaluable for improving immunotherapy approaches. Passive immunization strategies have shown great promise in pre-clinical models. Studies have shown that FITC-labeled antibodies can not only cross the blood-brain barrier [21] but also enter neurons and bind intracellular tau both *in vivo* and *ex vivo* [10,50]. In fact, these internalized antibodies co-localize with pathological tau markers inside the neurons containing tau pathology [20], and can prompt the clearance of intracellular tau aggregates through the endosomal/lysosomal system [51,52]. Recent advances in tau biology have shown that tau can exit neurons and propagate throughout the brain [53], suggesting that tau immunotherapy may work on both internal and external tau epitopes [12,13,54]. By defining the most potent immunogens within the tau protein and showing that the genetic environment of the host can lead to distinct immunogen profiles, new tools may be designed to better understand these mechanisms as well as improve the potential efficacy of passive immunization strategies that are rapidly progressing in the clinic.

## Conclusions

The present study provides the immunogenic profile to two different tau species in both wild-type and tau over-expressing mice, a tool that could be useful for the tau immunotherapy field moving forward. It also provides further support to the growing evidence that tau immunotherapy can effectively reduce tau pathology and neuroinflammation. Interestingly, one of the most immunogenic epitopes (amino acids 21-27) is conserved only in primates and humans, and this region has previously been linked to tau pathogenesis. Thus, perhaps this region contributes to disease pathogenesis through this previously unknown mechanism.

## Additional files

Additional file 1: Table S1. Complete list of identified tau immunogenic epitopes. The peptide sequences in tau proteins were detected as immunogenic motifs based on their florescence intensity in the array (threshold cut off ≥10,000 relative intensity). The residues identified by two independent experiments are labeled as *x2*. Additional file 2: Table S2. The peptide sequence and residue positions of immunogenic tau. Peptide sequence (red) corresponding to top ten immunogenic peptide motifs displaying the highest fluorescence intensity in the spot array.

## Abbreviations

AD: Alzheimer’s disease
ELISA: enzyme-linked immunosorbent assay
GFAP: glial fibrillary acidic protein
GSK3: glycogen synthase kinase 3
HRP: horse radish peroxidase
Ig: immunoglobulin
IFN: interferon
MAPK: mitogen activated protein kinase
MARK: microtubule affinity regulating kinase
PBS: phosphate-buffered saline
Wt: wild-type
